# Topically Applied Bacteriophage to Control Multi-Drug Resistant *Klebsiella pneumoniae* Infected Wound in a Rat Model

**DOI:** 10.3390/antibiotics10091048

**Published:** 2021-08-27

**Authors:** Mohamed S. Fayez, Toka A. Hakim, Mona M. Agwa, Mohamed Abdelmoteleb, Rania G. Aly, Nada N. Montaser, Abdallah S. Abdelsattar, Nouran Rezk, Ayman El-Shibiny

**Affiliations:** 1 Center for Microbiology and Phage Therapy, Zewail City of Science and Technology, Giza 12578, Egypt; p-mfayez@zewailcity.edu.eg (M.S.F.); p-abdallah.abdelsattar@zewailcity.edu.eg (A.S.A.); p-nrezk@zewailcity.edu.eg (N.R.); 2 Faculty of Biotechnology, October University for Modern Sciences and Arts, Giza 11223, Egypt; toka.abdelhakim1@msa.edu.eg (T.A.H.); nada.nasser2@msa.edu.eg (N.N.M.); 3 Department of Chemistry of Natural and Microbial Products, Pharmaceutical and Drug Industries Research Division, National Research Centre, Dokki, Giza 12622, Egypt; magwa79@gmail.com; 4 Department of Botany, Faculty of Science, Mansoura University, Mansoura 35516, Egypt; dmotelb87@gmail.com; 5 Department of Surgical Pathology, Faculty of Medicine, Alexandria University, Alexandria 21131, Egypt; rgm2006isa@yahoo.com; 6 Center for X-ray and Determination of Structure of Matter, Zewail City of Science and Technology, Giza 12578, Egypt; 7 Faculty of Environmental Agricultural Sciences, Arish University, Arish 45511, Egypt

**Keywords:** Gram-negative, *Klebsiella pneumoniae*, antibiotics, multi-drug resistance (MDR), bacteriophage, phage therapy, wound healing, in vivo, phage isolation, phage characterization, bioinformatics, wound infection, immunohistochemical (IHC)

## Abstract

(Background): Multi-drug-resistant *Klebsiella pneumoniae* (MDR-KP) has steadily grown beyond antibiotic control. Wound infection kills many patients each year, due to the entry of multi-drug resistant (MDR) bacterial pathogens into the skin gaps. However, a bacteriophage (phage) is considered to be a potential antibiotic alternative for treating bacterial infections. This research aims at isolating and characterizing a specific phage and evaluate its topical activity against MDR-KP isolated from infected wounds. (Methods): A lytic phage ZCKP8 was isolated by using a clinical isolate KP/15 as a host strain then characterized. Additionally, phage was assessed for its in vitro host range, temperature, ultraviolet (UV), and pH sensitivity. The therapeutic efficiency of phage suspension and a phage-impeded gel vehicle were assessed in vivo against a *K. pneumoniae* infected wound on a rat model. (Result): The phage produced a clear plaque and was classified as *Siphoviridae*. The phage inhibited KP/15 growth in vitro in a dose-dependent pattern and it was found to resist high temperature (˂70 °C) and was primarily active at pH 5; moreover, it showed UV stability for 45 min. Phage-treated *K. pneumoniae* inoculated wounds showed the highest healing efficiency by lowering the infection. The quality of the regenerated skin was evidenced via histological examination compared to the untreated control group. (Conclusions): This research represents the evidence of effective phage therapy against MDR-KP.

## 1. Introduction

*Klebsiella pneumoniae (K. pneumoniae)*, is a non-motile, encapsulated, Gram-negative bacteria that belongs to the *Enterobacteriaceae* family [1]. It is mainly responsible for primary severe community-onset infections, including hepatic abscess, necrotizing pneumonia, and nosocomial infections of the urinary tract, respiratory tract, wounds, and bloodstream [2]. *K. pneumoniae*, is one of the most common bacterial pathogens responsible for hospital-acquired bacterial infections [3]. *K. pneumoniae* infections are often considered one of the most fatal life-threatening infections to infants, older people, and immunocompromised patients as they are linked to both high morbidity and mortality rates [4].

Despite the evolution of broad-spectrum antibiotics, more than 50% of the mortality rates have been recorded for those infected with *K. pneumoniae* [5]. That was due to the worldwide expansion in multi-drug-resistant, *K. pneumoniae* strains, namely extended-spectrum β-lactamase (ESBL), produced and carbapenem-resistant strains, which render treatment more complicated. Moreover, the evaluated pathogenicity and the virulence factor caused by the development of capsules, mainly for hypervirulent strain, lipopolysaccharide (LPS), and fimbriae leaving only restricted choices for clinical treatment [6,7]. The World Health Organization (WHO) estimates that drug-resistant bacterial infections could kill approximately 10 million people per year by 2050 [8]. 

The skin is the largest organ in the body, which acts as a physical barrier protecting it from being attacked by pathogenic organisms, or toxic materials [9]. However, the skin solidity can be compromised by cuts (wounds) or burns at which the subcutaneous tissue is exposed to the surrounding environment, becoming more susceptible to microbial infection. As the proliferation of microorganisms increases, the invasion of deeper tissues begins [10]. When pathogens enter the lymphatic and blood vessels, sepsis develops [11], and sometimes, it leads to death. Wound infection, especially nosocomial infections, has a high mortality rate, affecting numerous people around the world each year [12]. As a result of increasing the expansion of disease correlated with MDR-KP bacteria, the riskiness of the burden has aggravated, and the hunt for alternatives to antibiotics is ongoing [13].

The conventional therapy for infected wounds, comprises excessive washing with saline, debridement of necrotic cells, and finally using an adequate therapy to minimize the microbial infection such as local or systemic antibiotics [14]. It was previously reported that topically applied antibiotics or other therapies are more effective than systemic administration for the remediation of contaminated wounds. In the case of systemic antibiotics, especially intravenous, the antibiotics did not attain the site infection in an appropriate quantity due to the lowering of circulation in the burned skin and the disability to destroy biofilm infections [15]. Moreover, it was mentioned before that topically applied antibiotics lead to the development of resistance so, the search for alternatives is demanding [16,17].

Therefore, the use of phage therapy to inhibit the growth of *Klebsiella* strains was applied [18,19]. Phages attack and kill bacteria via different mechanisms within their natural life cycles without interfering with human and mammalian cells [11,12]. To complete the lytic cycle, the phage must be adhered to a susceptible bacterial cell to infect its host. The cycle is accomplished by identifying and attaching to a particular receptor on the host cell’s surface. Adsorption to the host can take a place through any exterior structure depending on the phage tail structure and host receptor [20].

Phage has unique properties such as self-proliferation and high immunity with low side effects for eukaryotic cells [21,22]. Furthermore, it was characterized by its higher stability and efficiency in diverse environmental conditions against MDR pathogens, and it can be considered as a potential replacement for antibiotics [18,23]. The potential of utilizing phage therapy to overcome the crisis of MDR bacterial infections was proven in laboratory animal models. Thus, the current research describes the isolation and the characterization of *Siphoviridae* phage ZCKP8, then it evaluates its therapeutic potential in resolving *K. pneumoniae* mediated wound infection in rats.

## 2. Results

### 2.1. Bacterial Characterization

The Polymerase Chain Reaction (PCR) was confirmed that the twenty isolated *K. pneumonia*, including KP/15 strain by a 130 bp band corresponding to the conserved region in 16S-23S internal transcribed spacer region of *K. pneumoniae*. A partial-genome sequencing of KP/15 isolate was performed afterward (GenBank Acc. No. MZ504992). BLASTN of the 16S rRNA sequence had a 99% sequence identity to *K. pneumoniae* strain DSM 30104 16S ribosomal RNA (GenBank Acc. No. NR_117683.1).

### 2.2. Antibiotic Sensitivity Profile

The bacterial susceptibility to antibiotics representative of those administered to humans is classified into three criteria which are sensitive, intermediate, and resistant. The results demonstrated that *K. pneumoniae* isolates showed different sensitive responses to antibiotics classes, as it will be clearly shown in Table 1. The data indicated that six isolates were resistant to each of the Gentamicin and Imipenem, respectively, and thirteen isolates were resistant to each of the Aztreonam and Cefepime, respectively. In addition, the data revealed that eight, nine, eleven, twelve, and another twelve isolates were resistant to Amikacin, Tazobactam, Levofloxacin, Ceftazidime, and Tigecycline, respectively. Additionally, the data clarified that fifteen isolates were resistant to Ciprofloxacin and twenty *K. pneumoniae* strains were resistant to Sulfamethoxazole/Trimethoprim and Linezolid. KP/15 isolate and KP/1, KP/5, KP/6, KP/8, KP/9, KP/10, KP/11, KP/12, and KP/13 isolates are found to be MDR bacteria since they were resisting eight different antibiotics.

### 2.3. Morphology of ZCKP8 Phage

**Figure 1:**
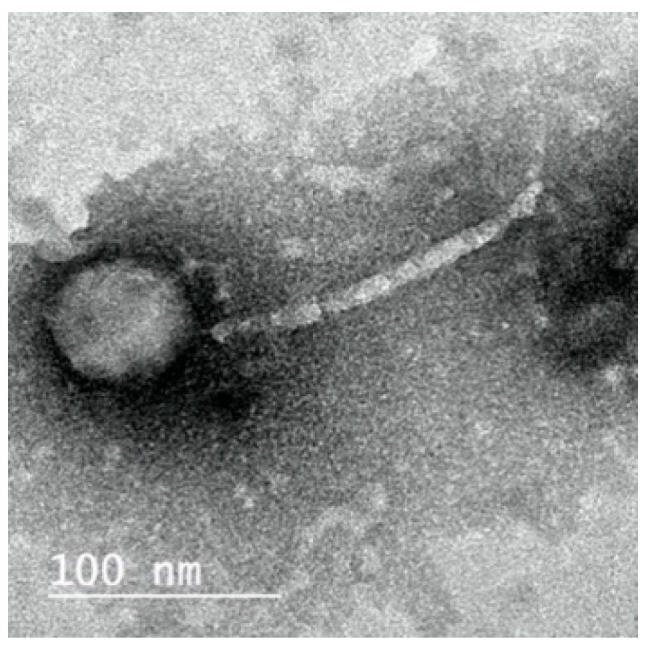
Transmission electron microscopic image of phage ZCKP8.

Electron microscopy revealed that ZCKP8, had an icosahedral head, filamentous, cross-banded, and non-contractile tail, which is typical of phages belonging to the family of Siphoviridae (Figure 1). The proportions of the phage head and tail length were also typical of the Siphoviridae with the head size being 55 nm while the tail length was 142 nm.

### 2.4. Phage Genome

**Figure 2:**
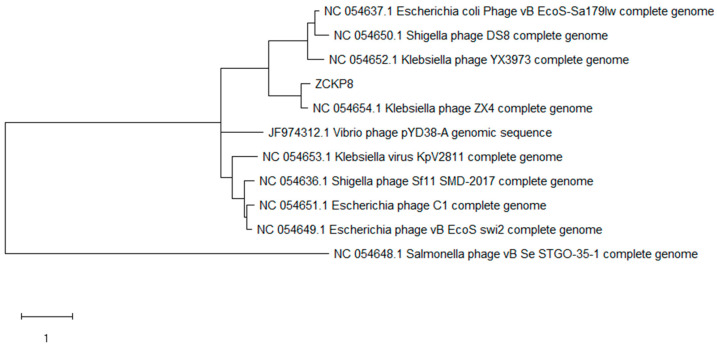
Phylogenetic relationships between ZCKP8 phage and BLASTN top-matched phages. The closely related phage is *Klebsiella* phage ZX4 (GenBank Acc. No. NC 054654.1).

**Figure 3:**
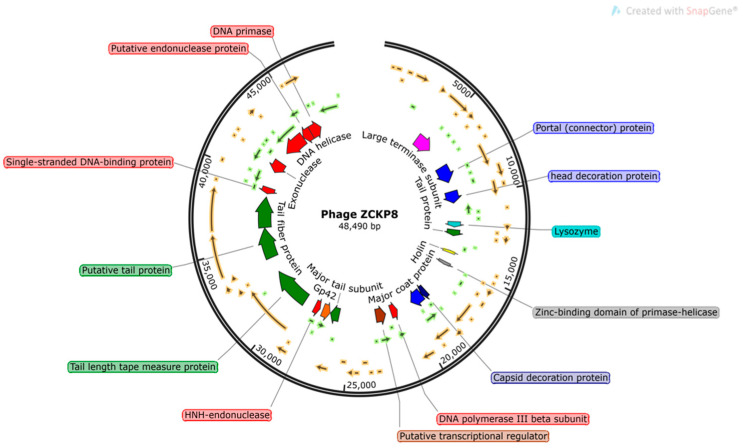
The ZCKP8 genome map. Only CDSs with known functions are labeled in their positions. Those representing hypothetical proteins are not labeled.

The phage ZCKP8 contains a double-stranded DNA genome estimated to be 48.5 kbp by Pulse Field Gel Electrophoresis (PFGE), which is comparable to the values indicated by the International Committee on Taxonomy of Viruses (ICTV) for phages that belong to the Siphoviridae family. The complete genomes of phage ZCKP8 were sequenced and deposited in the GenBank database (GenBank Acc. No. MZ440881). ZCKP8 has a DNA of 48.490 bp with an overall G + C content of 47.5%. BLASTn alignment and phylogenetic tree showed that Klebsiella phage ZX4 (GenBank Acc. No. NC 054654.1) was closely related to the genome of ZCKP8 as it will be shown in Figure 2. BLASTn analysis confirmed that ZCKP8 is a member of the Siphoviridae family, in the order Caudovirales. Fifty-three ORFs on the leading strand and thirty-four ORFs on the complementary strand were predicted through ORFfinder and PHASTER. The genomic map highlights functional genes that will be represented in Figure 3. Eighty-seven putative protein-coding genes were identified and among them there were twenty-six proteins that have assigned functions such as structural proteins, cell lysis proteins, DNA replication/transcription/repair proteins, and DNA packaging proteins. The list of the putative protein-coding genes was manually curated and listed in Supplementary Table S1. ZCKP8 phage does not encode any lysogenic phage-related proteins, such as transposases or integrase.

### 2.5. Phage Host Range and Efficiency of Plating (EOP) 

The host range of phage ZCKP8 was evaluated against bacteria that were isolated from clinical patients. The ZCKP8 phage was capable of producing lysis zones (≥20 plaques) on 8 out of 20 *K. pneumoniae* isolates, which showed that phage ZCKP8 was effective against *K. pneumoniae* isolates. A range of EOP for ZCKP8 phage was observed against different isolates of *K. pneumoniae* (Table 2).

### 2.6. Determination of the Bacteriophage Insensitive Mutant (BIM) Frequency

The BIMs were performed in vitro to determine the long-term effectiveness of phage ZCKP8 using a Multiplicity Of Infection (MOI) 100 of the bacterial host (KP/15) with phage ZCKP8 at 37 °C, where the BIM frequencies were 0.00521 ± 0.0029.

### 2.7. In Vitro Characterization of Phage ZCKP8

**Figure 4:**
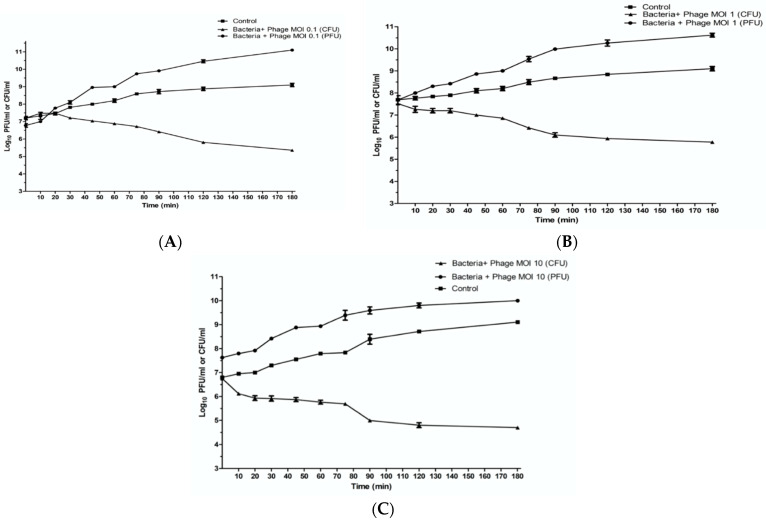
These plots represent the in vitro activity of phage ZCKP8 at 37 °C. These panels show bacterial counts and phage titers of *K. pneumoniae* KP/15 infected with ZCKP8 at (**A**) MOI of 0.1; (**B**) MOI of 1; (**C**) MOI of 10. MOI: Multiplicity of infection; CFU: Colony-forming unit; PFU: Plaque forming unit.

The infection and lysis characteristics of phage ZCKP8 were estimated at different MOIs (0.1, 1, and 10) throughout three hours in TSB broth to quantify its antibacterial lytic activity. The results showed that K. pneumoniae KP/15 was lysed by phage ZCKP8 with different ratios. The lysis characteristics, including time to lysis and burst size, were quantified over the period of infection to the onset of lysis for each MOI, with an MOI of 10 reducing viable bacteria from 6.7 log10 Colony-forming unit (CFU)/mL to below the limit of detection (2 log10 CFU/mL) at 37 °C during the three hours of the experiment that will be shown in Figure 4C. Log10 reduction in bacterial counts compared to the control were observed to be recorded between 80 and 90 min for both MOIs 1 and 0.1 as they will be shown in Figure 4A,B, and decreased dramatically below the detection limit after 120 min. Particularly, the bacterial populations did not recover within the 3 h course of the experiment. Under these circumstances, the reductions in the bacterial count were not accompanied by a measurable rise in phage titer. Phage replication was observed at lower MOI, which coincided with the commencement of the fall in the viable count. The data suggest that ZCKP8 is potent at the three tested MOIs and could control the bacterial growth over the three hours.

### 2.8. Phage Temperature, pH, and UV Stability

**Figure 5:**
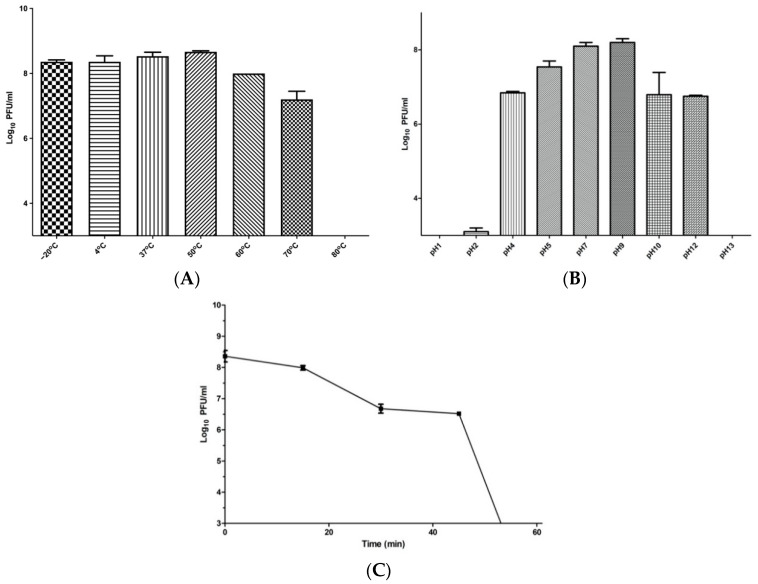
These diagrams represent the stability of phage ZCKP_8_ at different temperatures (**A**), at different pH values (**B**), and under UV lamp (**C**). PFU: Plaque forming unit.

The stability values of phage ZCKP8 were investigated for a whole one hour at different temperatures, pH, and UV which will be shown in Figure 5. Phage ZCKP8 titers were stable at approximately $10^{8}\;$ PFU/mL for 60 min at temperatures of −20, 4, 37, 50, and 60 °C. However, when the phage was incubated at 70 °C, its titer rapidly decreased to $10^{7}$ PFU/mL, and upon incubation at 80 °C, the titer drastically dropped below the detection limit. These results indicated that the phage could tolerate standard temperature environments, as it will be shown in Figure 5A. Phage ZCKP8 maintained an excellent pH range of 5.0–9.0 at approximately $10^{10}$ PFU/mL, but its titers dramatically decreased ten times at pH (4.0, 10.0, and 12). When incubated at pH 1 and pH 13, the phage was utterly inactive. Thus, the optimum pH range for phage ZCKP8 was found to be 5.0–9.0, as it will be shown in Figure 5B. Phage ZCKP8 maintained an excellent activity of the UV stability even when it decreased from $10^{10}$ to approximately $10^{8}$ PFU/mL after 15 min. However, its titers dramatically decreased 10 folds after 30 min, and the phage was still stable within 45 min. After 45 min, the titers of phage gradually decreased until it was utterly inactive at 60 min. Thus, the optimum UV stability for phage was observed at 15, 30, and 45 min, as shown in Figure 5C.

### 2.9. In Vivo Wound Healing

#### 2.9.1. Photodocumentary Analysis and Wound Healing Percentage

**Figure 6:**
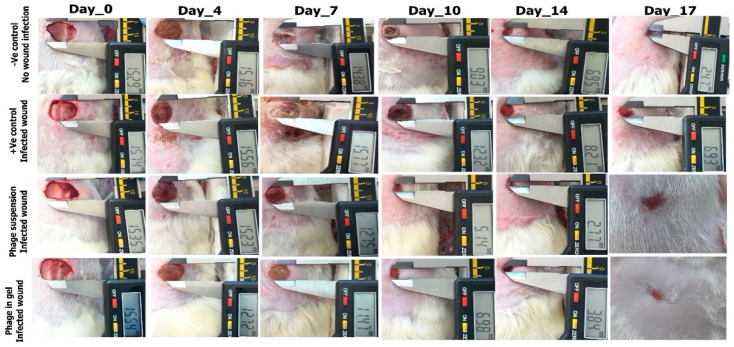
Morphometric examination of full-thickness excision wounds in rats at a fixed focal distance by Millimetre (mm) using digital photographs.

The images shown in Figure 6 represent the treated and nontreated wounds with the phage on zero day, 4th, 7th, 10th, 14th, and 17th post-surgery.

#### 2.9.2. Wound Healing Percentage

**Figure 7:**
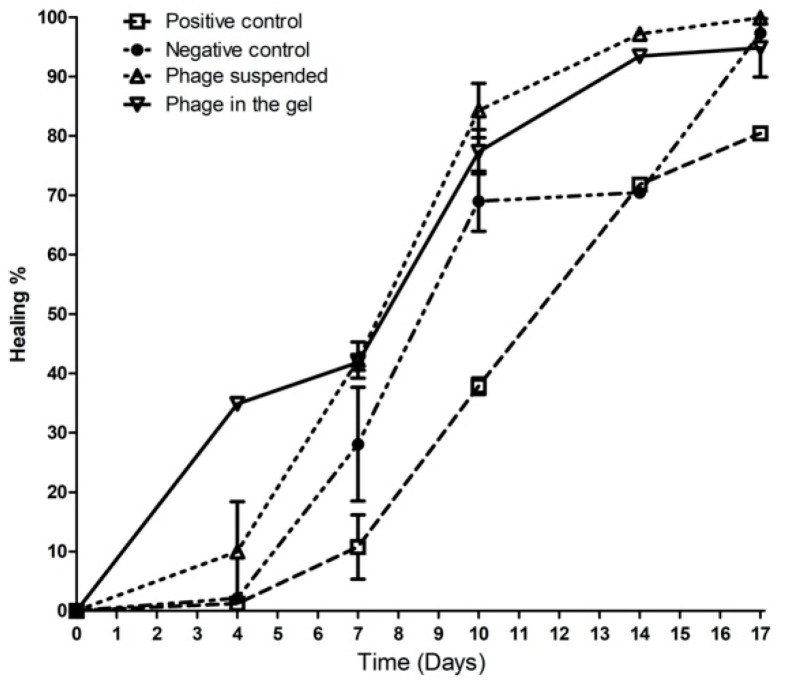
The wound healing percentage from day 0 to the 17th day in the four groups.

Starting from the 7th day, the two treated infected groups with phage suspension and phage impeded in gel showed a reduction in the wound area, where the wound closure percentages were 32.42% and 37.84%, respectively. While, the wound healing percentage was 15.3% and 0% in the negative control group and the positive control group (Infected wound without treatment), respectively. On the 17th, the treated groups with phage suspension and gel reached a maximum of 99% of wound closure, where the negative control group reached 97.43% while, the positive control group showed a minimum of 79.76% wound closure, as shown in Figure 7.

#### 2.9.3. Histopathological Analysis of Wound Healing

**Figure 8:**
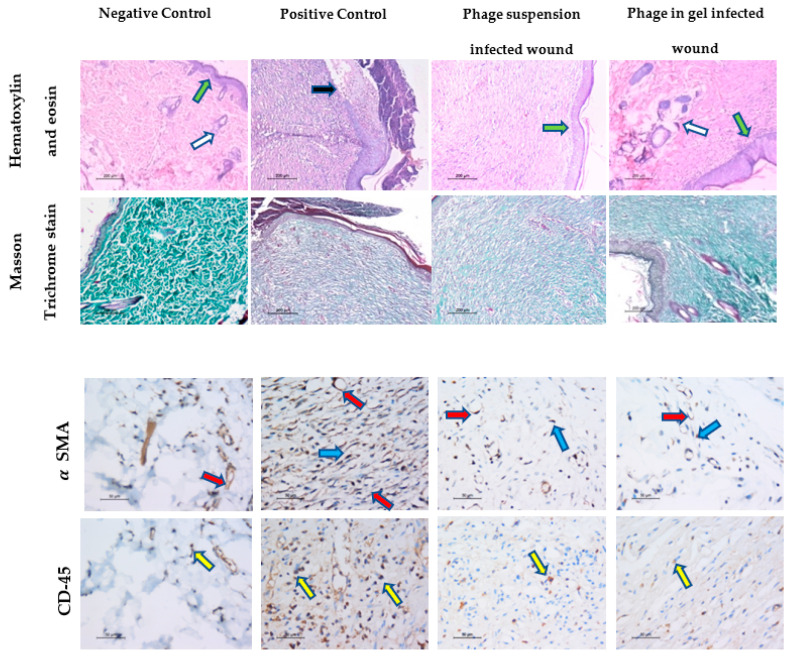
Histological analysis of wounded skin section from different rat groups at the 17th-day post wounding. Hematoxylin and eosin (H&E) staining, ×100, Masson Trichrome stain, ×100 and IHC by using α-SMA, and CD-45, ×400 showed in the non-infected, non-treated group complete skin wound healing and remodeling, the infected non-treated group shows epidermal ulceration and crust formation, early inflammatory phase of wound healing and a minimal amount of fibrosis, denoting delayed wound healing. While the infected groups treated with phage suspension and phage embedded in gel show, a complete wound healing and remodeling with adequate amount of fibrosis and epidermal re-epithelialization in the latter group denoting complete maturation of the healing process.

The histopathological images represented in Figure 8 show that the wound tissues from day 17 are stained with H&E, Masson’s trichrome, and immunostaining.

The skin section from the positive and negative control groups showed epithelial re-epithelialization, with an epidermal thickness of 47 ± 1.0 µm, and regenerated skin appendages >5/HPF (score 3). The dermis showed a remodeling phase of healing formed of collagen fibers mean was 96 ± 0.8% of the wound area evidenced by Masson’s trichrome staining, with scant residual granulation tissue formed of few dermal lymphocytes mean was 7 ± 1.1/HPF using CD45 IHC. The mean blood vessel count was 4 ± 0.7/HPF and 5 ± 2.1 myofibroblasts/HPF using α-SMA IHC. 

The skin section from the nontreated infected group showed epidermal ulceration, with a thick crust of 33.6 ± 1.2µm, lacking skin appendages (score 0). The dermis showed fibrous tissue, evidenced by Masson trichrome staining and was predominantly in the inflammatory phase with early proliferation phase of wound healing consisting of abundant acute inflammation, granulation tissue amount formed of newly-formed blood vessels, mean 35.2 ± 1.3/HPF, the inflammatory cells mean was 80 ± 2.0/HPF, the myofibroblasts count mean was 15.3 ± 2.4/HPF. With few dermal fibrous tissue fibers, the mean was 22.3 ± 1.7% of the total area was confirmed by using α-SMA IHC, and the increased number of lymphocytes 60/HPF was confirmed by using immunohistochemistry for CD-45.

The skin section from the infected wound was treated with phage suspension, showing epidermal re-epithelialization of epidermis thickness 33 ± 1.2 µm, with the regeneration of the skin appendages <5/HPF (score 2). In addition, the dermis shows the remodeling phase of wound healing formed mainly of collagen fibers, mean area 75 ± 2.4% evidenced by using Masson’s trichrome staining, and the residual amount of granulation tissue formed of newly formed blood vessels, mean 6 ± 0.4/HPF, myofibroblasts 7 ± 0.9/HPF was confirmed by using immunohistochemistry for α-SMA. In addition, the mean many lymphocytes were 8 ± 1.1/HPF and confirmed by using immunohistochemistry for CD-45.

The skin section from the infected wound was treated with phage embedded in gel showed epidermal re-epithelialization with a mean thickness of 50.3 ± 0.9 µm with regenerated skin appendages >5/HPF (score 3). The healing process was in the remodeling healing phase; with the wound area formed mainly of collagen fibers evidenced by Masson’s trichrome staining, the mean area was 98.4 ± 1.3%. The dermis revealed the scanty amount residual of the granulation tissue which was fully matured into collagen fibers, the mean count of the newly formed blood vessels was 5 ± 0.4/HPF, and the mean number of myofibroblasts was 2 ± 0.4/HPF confirmed by using α-SMA IHC with decreased number of the lymphocytes, mean value was 6.3 ± 1.0/HP confirmed by using CD-45 IHC.

## 3. Discussion

The increased rate of multi-drug resistant *K. pneumoniae* poses a significant public health risk especially with the restricted number of therapeutic choices. Phages are promising alternative candidates to chemical antibiotics because of their high host specificity, natural abundance, and developmental characteristics for this task.

In this study, phage ZCKP8 was isolated from wastewater and produced lysis zones with halos (≥20 plaques) when plated on *K. pneumoniae* isolates. The results revealed that the phage ZCKP8 was efficiently generating 100% lysis in *K. pneumoniae* isolates. In comparison with phages vB_KpnS_FZ10, vB_KpnS_FZ41, vB_KpnP_FZ12, and vB_KpnM_FZ14, the phage demonstrated strong lytic activity, which indicates its potential for bacterial infection prevention and therapy. From the above-mentioned phages, three out of four investigated phages produced lysis sites with halos [24].This phenomenon was most likely caused by the release of soluble polysaccharide-degrading enzymes such as capsule depolymerase, which can possibly destroy the *K. pneumoniae* capsules [25]. 

Interestingly, ZCKP8 had shown to have a slight range when it infected 8 out of 20 bacterial strains. However, as demonstrated by ZCKP8, the phage can lyse several *Klebsiella* strains. Usually, the host is utilized to isolate the phage. The traditional isolation process aims to use the most susceptible bacterial host for enrichment Nevertheless, current evidence suggests that isolating phages with broader host ranges is more likely when numerous hosts are used during the isolation [26]. Furthermore, the phage’s host range activity can evolve and change over time [27]. In contrast, the lytic phage KPO1K2, which is specific for *K. pneumoniae* B5055, may infect a variety of *K. pneumoniae* strains as well as several *E. coli* strains, and hence has a broader host range than the clade-specific phage BO1E [28].

Interestingly, in the temperature range from −20 and 70 °C, it was observed that ZCKP8 was relatively thermally stable. ZCKP8 maximal activity was detected at 50 °C, with a moderate decrease of 2 log_10_ PFU/mL at 70 °C. *K. pneumoniae* phages, including wKp-lyy15, phage Z, PKP126, vB_Klp_1, vB_Klp_3, vB_Klp_4, vB_Klp_5, and vB_Klp_6, were previously mentioned to be active at 70 °C [29,30,31].

In this current study, phage ZCKP8 maintained a high activity in the pH range of 5.0–9.0. While in other studies the titer of the phage was dramatically decreased to 1 log_10_ at pH 4.0 and 10.0 The ZCKP8 phage was stable at pH 7 such as the previously tested Phage vB-GEC_Ab-M-G7 [32], but PFU/mL reduced two logs at pH 5, one log at pH 9, three logs at pH 11, and over five logs at pH 3 after 24 h of incubation at 37 °C. Phage ZCKP8 maintained an excellent activity of the UV stability around 10^8^ PFU/mL at 15 min, but the phage’s titer rapidly declined until it was entirely inactive at 60 min. As a result, the best UV stability for phage was found at 15, 30, and 45 min. The titer of phage decreased considerably by almost 80% after 10 min of UV exposure, and after 60 min of exposure, the viability was fewer than 0.0007% [33]. 

This research tested the antibacterial efficacy of phage ZCKP8 both in vitro and in vivo on a rat model. The replication dynamics findings showed that phage ZCKP8 successfully reduced the growth of *KP*/*15*, with negligible resistant colonies developing at MOI 0.1 due to a low rate of resistance to ZCKP8’s lytic activity. Furthermore, at MOI 1 and 10, phage ZCKP8 lysed *KP/15*, resulting in a reduction in viable bacteria as the PFU increased to 10.0 log_10_ PFU/mL. The results showed that the efficacy of phage ZCKP_8_ to control the bacterial host (*KP*/*15*) is concentration-dependent, which matches with the results from phage vB_KpnS_Kp13. That is because the phage vB_KpnS_Kp13 prevented the propagation of *K. pneumoniae* 533 in a concentration-dependent manner in LB broth over 24 hours at an MOI between 0.0001 and 100 [34]. By using a high phage titer, the chances of phage to attack its host cells with high efficacy in killing and reducing the number of target hosts will increase over a short period.

It was reported that several phages were isolated to treat *K. pneumoniae* and most of them belong to *Siphoviridae* [35], *Podoviridae* [36], and *Myoviridae* [24]. Moreover, the genome size of the isolated ZCKP8 was 48.5 kbp and the images of TEM confirmed that this phage belongs to the *Siphoviridae* family. Bioinformatic predictions demonstrated that ZCKP8 is a virulent phage as protein-coding genes related to lysogenies such as transposases, integrase, and λ repressor protein (CI) were not identified [35,36,37]. Further experimental testing will be performed to confirm the absence of virulence genes. Therefore, this phage may be safe for phage therapy application due to the absence of the antibiotic resistance genes and the genes that encode for virulence factors [38]. 

In this research, the therapeutic efficiency of phage ZCKP8 was examined on rats to treat full-thickness wounds infected with *K. pneumoniae* clinical isolate (*KP/15*) which is resistant to multiple antibiotics, including Amikacin (AK; 30 μg), Aztreonam (ATM 30 μg), Ciprofloxacin (CIP; 5 μg), Ceftazidime (CAZ; 30 μg), Cefepime (Fep; 30 μg), Gentamicin (CN; 10 μg), Imipenem (IPM; 10 μg), Levofloxacin (LEV; 5 μg), Sulfamethoxazole/trimethoprim (SXT; 25 μg), Tigecycline (TGC; 15 μg), piperacillin/tazobactam (TZP; 110 μg), and Linezolid (LZD; 30 μg). From the histopathological results, it was clear to us that among the groups, whose wound was infected with *K. pneumoniae*, the better healing was observed in the group treated with the phage embedded in the gel. This was evidenced via the complete healing process, reaching the remodeling phase with full restoration of the epidermal thickness and skin appendages regeneration. We found this comparable to the sterile wound healing process for the nontreated and noninfected group with some superior features such as the epidermal thickness, the collagen deposition and the minimal amount of the residual granulation tissue [39]. The better healing efficiency for this group might be attributed to the longer residence time of the phage at the infected wound site, which allows delivering an enormous dose of phage capable of overcoming the bacterial infection. On the other hand, the phage solution revealed essential features of the advanced healing process, the latter was not as fully mature as using the phage embedded in the gel. This could be attributed to the easy elimination from the wound site, so the amount of phage delivered to the infected wound site is not accurate and may not be able to overcome all the bacterial infection quickly [40]. 

The biofilm genesis via bacterial infection was reported to be participating in the extended inflammation and pathologic mechanisms responsible for delayed wound healing, as in the nontreated infected group. The findings of this study show that topical application of phages represents a potentially effective treatment for wounds infected with *K. pneumoniae* clinical isolate that may enhance the healing process due to their potent antimicrobial activity and disruption of biofilm formation [41]. Therefore, this phage can minimize the incidence of septicemia caused by wound infection and decrease mortality in patients.

A phage diversity understanding in nature can contribute to the future development of phage-based therapeutics. Phage treatment has shown a promising possible therapy to MDR-KP’s continuing growth and dissemination. Furthermore, the widespread use of phage therapy in Eastern Europe and the outcomes of the few human studies conducted in the West suggest that phages are usually deemed safe for use in humans.

## 4. Materials and Methods

### 4.1. Bacterial Strains Isolation and Stocking

In March 2019, clinical sputum samples from *K. pneumoniae* patients were collected from hospitals and provided to Zewail City of Science, Technology, and Innovation’s center for microbiology and phage therapy. Sputum samples were inoculated aseptically on MacConkey agar (MAC; Oxoid, UK) plates and incubated at 37 °C overnight. A total of twenty *K. pneumoniae* isolates were recognized by their morphology and were preserved at −80 °C in tryptone soy broth, known as (TSB), that includes (*w*/*v*) 20% glycerol. The isolated colonies of the *K. pneumoniae* bacteria were cultured at 37 °C after overnight growth on the tryptic soy agar, short for (TSA).

### 4.2. Bacterial Identification and Confirmation by PCR

PCR reaction was performed using 25 μL of 1× Master Mix (Thermo Fisher Scientific, Waltham, MA, USA), 1 µL of the forward primer (5′-ATTTGAAGAGGTTGCAAACGAT-3′), 1 µL of the reverse primer (5′-TTCACTCTGAAGTTTTCTTGTGTTC-3′), and 5 μL of crude DNA extract (isolated via QIAamp DNA Mini Kit), and 18 μL of nuclease-free water. PCR conditions were as follows: initial denaturation at 94 °C for 5 min, then 35 cycles at 94 °C for 1 min, 58 °C for 1 min, and 72 °C for 1 min. The final extension was at 72 °C for 7 min. PCR products were separated by agarose gel electrophoresis in a 1.5% (*w*/*v*) gel. The band was supposed to be found at 130 bp [42].

### 4.3. 16S rRNA Gene Sequencing

A second PCR reaction was carried out in 50 μL volumes of the following; 25 μL of 1× Master Mix (Thermo Scientific), 5 μL of crude DNA extract (isolated via QIAamp DNA Mini Kit), and 18 μL of nuclease-free water, and 1 µL of the forward primer (5′-AGAGTTTGATCCTGGCTCAG-3′), 1 µL of the reverse primer (5′-TACGGYTACCTTGTTACGACTT-3′). PCR conditions were as follows: initial denaturation at 94 °C for 3 min, then 30 cycles at 94 °C for 30 s, 55 °C for 30 s, and 72 °C for 1 min. The final extension was at 72 °C for 10 min. PCR products were separated by agarose gel electrophoresis in a 1.5% (*w*/*v*) gel [43]. The amplified fragment of 16S rRNA gene was purified using QIAEX II Gel Extraction Kit (QIAGEN, Hilden, Germany) following the manufacturer’s instructions [44]. The purified PCR product was sequenced along with the aforementioned universal primer set. The 16S rRNA sequence was determined with a model 373A automated fluorescent DNA sequencer (Applied Biosystem, Waltham, MA, USA). The obtained nucleotide sequence of 16S rRNA gene was processed through Finch TV software (https://digitalworldbiology.com/FinchTV/ accessed on 27 June 2021). BLASTN (Basic Local Alignment Search Tool, http://www.ncbi.nlm.nih.gov/BLAST/Blast.cgi/, accessed on 27 June 2021) against the 16S ribosomal RNA database was performed to identify the isolated strain [45]. The sequenced 16S rRNA sequence was deposited into the GenBank under the accession number SUB9914464.

### 4.4. Antibiotic Sensitivity Test

In accordance with the National Committee for Clinical Standards guidelines, antimicrobial sensitivity testing was performed on 20 *K. pneumoniae* isolates using disk diffusion methods. By evaluating the bacterial growth inhibition zone around the antibiotic discs, twelve different antibiotics were chosen based on their effectiveness against the *K. pneumoniae* bacteria, belonging to the Enterobacteriaceae family. Each strain was exposed to different discs of the antibiotics such as Amikacin (AK;30 μg), Aztreonam (ATM 30 μg), Ciprofloxacin (CIP; 5 μg), Ceftazidime (CAZ; 30 μg), Cefepime (Fep; 30 μg), Gentamicin (CN; 10 μg), Imipenem (IPM; 10 μg), Levofloxacin (LEV; 5 μg), Sulfamethoxazole/trimethoprim (SXT; 25 μg), Tigecycline (TGC; 15 μg), piperacillin/tazobactam (TZP; 110 μg), and Linezolid (LZD; 30 μg).

### 4.5. Phage Selection, Isolation, Purification, and Amplification

Six different phages were obtained from sewage water in Giza, Egypt. The water samples were centrifuged at 4000 rpm and the supernatant was filtered from other bacteria using 0.2 µm porous syringe filters [46]. Enrichment was performed to isolate the phages, then chloroform was added for phage filtration [47]. The purified culture was centrifuged for 20 min at 5000 rpm and 4 °C. The antibacterial effectiveness of isolated phages was assessed by carrying out the double-layer agar experiment and the spotting test [48]. A clear plaquing phage was selected for further characterization using *K. pneumoniae* (*KP*/*15*) as a bacterial host. Each phage plaque was purified by repeating the isolation of a single plaque process. All isolated phages were amplified in TSB, and the lysates were centrifuged at 5000× *g* for 15 min at 4 °C [49,50]. After that, the supernatant, containing phages, was centrifuged for 1 h at 15,300× *g* at 4 °C. The pellet was resuspended in SM buffer and purified through 0.22 μm syringe filters. Moreover, double-agar overlay plaque assays were used to assess the titers of the phages [51]. The titer of phage suspension was diluted 10 folds in a 96-well plate, where each lane contained exactly 180 μL of SM buffer and 20 μL of phage suspension. Only 10 μL aliquots were spotted in triplicate onto bacterial lawns [52].

### 4.6. Characterization of the Isolated Phage

#### 4.6.1. Pulsed Field Gel Electrophoresis (PFGE)

Pulsed-field gel electrophoresis was used to determine the genomic size of phage ZCKP8 (10^10^ PFU/mL) DNA [53]. In brief, phage was suspended in agarose plugs and digested with lysis buffer (0.2% *w*/*v* SDS [Sigma Aldrich, Gillingham, UK]; 1% *w*/*v N*-Lauryl sarcosine [Sigma Aldrich, Gillingham, UK]; 100 mM EDTA; 1 mg/mL Proteinase K [ThermoFischer Scientific], Waltham, MA, USA), overnight at 55 °C for 18 h with gentle shaking to lyse the phage capsids and digest protein components. Two slices of agarose-containing DNA were inserted into the wells that contain 1% *w*/*v* agarose gel after being washed with washing buffer. The gel was run in 0.5× Tris-borate-EDTA at 14 °C for 18 h at 200 V (6 V/cm) with a switch time of 30 to 60 s by using a Bio-Rad CHEF DRII system. The genome size was determined in comparison with standard concatenated lambda DNA markers (Sigma Aldrich, Gillingham, UK).

#### 4.6.2. Examination of Phage Morphology by Electron Microscopy (TEM)

Phage ZCKP8 morphology was imaged on glow-discharged (1 min under vacuum) by using TEM via the National Research Center (Cairo, Egypt) [54]. The phage suspension was submerged on Formvar carbon-coated copper grids (Pelco International); the phage was fixed with glutaraldehyde (2.5% *v*/*v*), washed, and stained with 2% phosphotungstic acid (pH 7.0). A transmission electron microscope was used to examine grids after they had dried (JEOL 1230).

#### 4.6.3. Host Range Determination

The host range of six different phages isolated from sewage water, including phage ZCKP8, tested over twenty *K. pneumoniae* isolates. An aliquot of 100 μL of freshly cultured bacteria was mixed with 4 mL of 0.5% top agar and poured onto a 1.5% TSA agar plate for the spot examination. After the top agar had solidified, 10 μL of phage suspension were spotted on the plates and incubated overnight at 37 °C [2]. 

#### 4.6.4. Phage DNA Sequencing

Proteinase K treatment (100 μg/mL in 10 mM EDTA pH 8) was used to prepare genomic DNA from phage ZCKP8 ($10^{10}$ PFU/mL) lysates, followed by resin purification using the Wizard DNA kit (Promega, UK), according to the manufacturer’s instructions. The Illumina MiSeq platform was used to perform DNA Sequencing. Library preparation was performed using the Illumina Nextera tagmentation protocol (Illumina, Cambridge, UK). The data contained paired-end sequences with a length of 150 bp. The sequences were evaluated for accuracy using FASTQC [55]. Sequences were de novo assembled using SPAdes [56] with K-mers of 21, 33, 55, 77, and 99 resulting in a unique contig of 48,490 bp. BLASTNagainst the nucleotide collection database was used to identify the matches to previously sequenced phages. Then, the top matched phages were imported into MEGA-X [57] to draw a phylogenetic tree using the CLUSTAL-W aligner [58] and the best Maximum Likelihood fit model (GTR: General Time Reversible substitution model, G: Gamma distributed among sites). Open-reading frames were predicted using NCBI ORF finder search server using Methionine and alternative initiation codons as a start codon. Then, the predicted ORFs were compared against the NCBI non-redundant protein sequences (nr) database using BLASTp to identify putative coding sequences (CDSs). In addition, the predicted ORFs and coding sequences were compared to those predicted through PHASTER [59]. The circular genomic map was prepared using SnapGene Viewer (GSL Biotech; available at https://www.snapgene.com/; access on 20 June 2021). The annotated complete genome of phage ZCKP8 was deposited in the GenBank database under the accession number of MZ440881.

#### 4.6.5. In Vitro Characterization of Phage ZCKP8

For the in vitro assay, Phage lysates were centrifuged, and the supernatant was used for testing lytic activity against MDR bacterial strain [48]. Lytic ability characteristics of phage ZCKP8 at different multiplicities of infection (MOI) of 0.1, 1, and 10 PFU/CFU was demonstrated on exponential-growth-phase cultures of KP/15 (10^8^ CFU/mL) in comparison to bacterial control at a temperature of 37 °C [60]. Colony-forming units (CFU) and plaque-forming units (PFU) were also estimated. Briefly, the bacterial culture at a given concentration (control) and phage matching the desired MOI (Test) was filled with two flasks. Phage ZCKP8 was added to *K. pneumoniae* KP/15 in the log-phase of growth. The concentrations of BS, CFU, and PFU were estimated periodically at different time intervals (0, 10, 20, 30, 40, 60, 75, 90, 120, 150, and 180 min). Experiments were performed in triplicates.

#### 4.6.6. Efficiency of Plating (EOP)

Phage ZCKP8 was tested overall the susceptible bacterial strains lysed in the spot assays three times using eight decimal dilutions [61]. (EOP = phage titer on target bacterium/phage titer on bacterium) to determine the efficiency of each phage against a diversity of target bacteria. Conditions of these experiments were the same as spot tests using log-phase bacteria. Therefore, 50 μL of each bacterial isolate was added to top agar, and various phage dilutions were spotted on TSA ager plates. The plates had been incubated at 37 °C overnight. EOP was calculated the next day as the average PFU on target bacteria divided by the average PFU on host bacteria.

#### 4.6.7. Determination of the Frequency of BIM

As previously described, the frequency of the emergence of BIM was estimated [62]. Phage ZCKP8 was combined with bacterial host strains that were confirmed to be susceptible to the phage, including *K. pneumoniae* strains, at a MOI of 100. After 10 min of incubation at 37 °C, the suspension was serially diluted and spotted using double-agar overlay plaque testing in triplicate; then, the plates were incubated overnight. BIM was calculated by dividing viable bacterial counts that remained after phage infection by initial viable counts.

#### 4.6.8. Phage pH, Temperature, and UV Stability

The temperature stability of phage ZCKP8 ($10^{10}$ PFU/mL) was assessed at −20 °C, 4 °C, 37 °C, 50 °C, 60 °C, 70 °C, and 80 °C after 1 h incubation in a water bath. The serial dilutions of the phage were spotted three times immediately after incubation, using a double-layer standard technique; on a lawn of host strain (KP/15) to estimate the titers of phage [63]. The UV stability of phage ZCKP8 was evaluated at 15, 30, 45, and 60 min. After the incubation, serial dilutions of phage were spotted in triplicate, using standard double-layer technique; on a lawn of host strain (KP/15) to estimate phage titers. The bacterial counts of ZCKP8 at different pH values of 1, 2, 4, 5, 7, 9, 10, 12, and 13 were determined after 24 h incubation, and then the phage titer was determined [64]. To preserve comparative conditions, different pH values were achieved in the SM phage buffer [65].

### 4.7. In Vivo Wound Healing Efficiency Using ZCKP8 Phage on a K. pneumoniae Infected Wound on a Rat Model

#### 4.7.1. Surgical Procedures of Full-Thickness Wound Model

The phage can be applied locally to the wounded skin area either suspended in SM buffer solution or impeded in a gel vehicle (KLY lubricating Jelly) to examine its healing potential on the *K. pneumoniae* infected full-thickness excision skin wound using a rat model. All in vivo animal studies were approved by the Animal Care Committee of the Alexandria University (ALEXU-IACUC), with an authorized acceptance for the surgical procedures (AU-IACUC-14/2100601-3-7).

Twenty Wistar male rats (8 weeks old) were chosen in the in vivo study weighing approximately 180–200 g. The rats were housed solely in independent stainless-steel boxes with free access to standard laboratory diet and mineral water ad libitum. The surgical steps for the complete thickness excision wound were achieved based on what was previously published [66,67,68]. The animals were doped by injecting xylazine (10 mg/kg) and ketamine (60 mg/kg) into the muscle. Then, the back hair was segregated using an electric shaver, and circular, 15-mm diameter full-thickness wounds were generated using surgical scissors (one wound/rat). Wound infection was accomplished by inoculating *K. pneumoniae* into the wound area (1.5 × $10^{8}$ CFU/mL) for two hours before applying the treatment. Ketoprofen (100 mg/kg) was given via I.M route immediately after surgery to alleviate pain till the symptoms were resolved. The % wound closure of each rat was estimated via measuring and photographing the reduction in the wound diameter. The animals were randomly assorted into four sections with five rats, including negative control group, applied sterile gauze (no wound infection). The three rest groups applied sterile gauze [69] phage suspension (200 µL) [70], and phage impeded in a KYL commercial Lubricating gel by mixing phage suspension (9.0 log_10_ PFU/mL) to gel at a ratio of 1:1, volume per volume (*v*/*v*) respectively to *K. pneumoniae* infected wound [71].The wound was rated and photographed via a digital camera on days 3, 7, 10, 14, and 17 after surgery [72]. The wound area was estimated using digital caliber, and the % wound closure rate was calculated from the following equation [73].

Wound closure (%) = [1 − (Wound area at the given day/Wound area at the day 0)] × 100.

#### 4.7.2. Histological Examination

On day 17 the rats were sacrificed, followed by removing the tissue from the wound bed and its surrounding healthy skin to assess the skin restoration in the healed region. Then, the tissue was fixed in 10% formaldehyde and embedded in paraffin blocks for histopathological investigations. After that, the skins sections were stained with hematoxylin and eosin (H&E) and examined under a light microscope (Leica, Wetzlar, Germany). Then, a histological examination of the whole wound area was carried out and the mean value of the percentage of the fibrous tissue, the epidermal thickness was quantified using ImageJ, v1.53 (Baltimore, MD, USA). The skin appendages were scored (no skin appendages 1, few <5/wound are 2 and >5/wound area 3). The skin sections were also stained with Masson Trichrome staining. ImageJ, color deconvolution v1.53 (Baltimore, MD, USA) was used to quantify the percentage area of fibrosis in each specimen [72,73].

#### 4.7.3. IHC Staining and Interpretation

IHC of all sections was carried out using the Avidin-Biotin-Peroxidase method [30]. CD45 (Ready to use primary antibody, mouse anti-human, monoclonal antibody, P0042; Leica Biosystems, Buffalo Grove, IL, USA) and α-SMA (Ready to use primary antibody, mouse anti-human, monoclonal antibody, P0943; Leica Biosystems, Buffalo Grove, IL, USA) were used to stain the lymphocytes, blood vessels, and myofibroblasts respectively. Then, the antibodies were added to each section using the Bond-Max fully automated immunostainer (Leica Biosystems, Buffalo Grove, IL, USA). The quantification of the IHC of CD45 and α-SMA was performed in each slide, using the quantitative-image analysis (Leica microsystems, Heerbrugg, Switzerland). At the end of the experiment, the concentration of phage was determined to ensure that the dose was fixed throughout the in vivo experiment.

## 5. Conclusions

The continuous increase in drug-resistant bacteria has led scientists to search for alternative treatments to antibiotics such as phage therapy. The illustrated results suggest that the phage ZCKP8 is a promising candidate for further investigation in phage-therapy research. In conclusion, a novel phage, ZCKP8, with activity against *K. pneumoniae* KP/15 was isolated and characterized in vitro. The phage belongs to the *Siphoviridae* family, and showed good tolerance to a range of temperatures, pH and UV, and was stable under long-term storage conditions. Additionally, ZCKP8 showed high therapeutic efficacy in vivo on the rat model as it treats full-thickness wounds infected with a *K. pneumoniae* clinical isolate, which is resistant to multiple antibiotics. Thus, Phage ZCKP8 showed excellent potential to be used as a therapeutic agent against *K. pneumoniae* infections. Therapeutic trials will be used to confirm its potential as an antibacterial therapeutic agent. Hopefully, in the future, the availability of a large variety of phages may allow the development of multi-phage cocktail therapy possible.

## Figures and Tables

**Table 1:** Antibiotic sensitivity profile for *K. pneumoniae* isolates over 12 different antibiotics.

Antibiotics	AK 30	ATM 30	CIP 5	CAZ 30	Fep 30	CN 10	IPM 10	LEV 5	SXT 25	TGC 15	TZP 110	LZD 30	MAR Index
KP/1	S	R	R	R	R	S	I	I	R	S	I	R	0.5
KP/2	S	R	R	R	R	S	S	I	R	R	R	R	0.7
KP/3	S	S	R	I	I	S	S	R	R	R	I	R	0.4
KP/4	S	S	I	I	I	S	I	I	R	R	I	R	0.3
KP/5	R	S	R	R	R	S	R	R	R	R	R	R	0.8
KP/6	R	R	R	R	R	S	S	I	R	R	R	R	0.8
KP/7	I	S	S	S	S	S	I	S	R	R	I	R	0.3
KP/8	R	R	R	R	R	R	R	I	R	R	R	R	0.9
KP/9	R	R	R	R	R	R	R	R	R	S	I	R	0.8
KP/10	R	R	R	R	R	R	R	R	R	R	R	R	1
KP/11	R	R	R	R	R	R	R	R	R	R	I	R	0.9
KP/12	S	R	R	R	R	R	S	R	R	R	I	R	0.8
KP/13	R	R	R	R	R	R	I	R	R	R	R	R	0.9
KP/14	S	I	R	I	I	S	S	R	R	I	I	R	0.3
KP/15	I	R	R	R	R	S	S	I	R	R	R	R	0.7
KP/16	R	R	R	I	S	S	R	R	R	S	R	R	0.7
KP/17	S	I	R	I	I	S	S	R	R	S	I	R	0.3
KP/18	S	R	I	S	R	S	S	S	R	S	S	R	0.3
KP/19	S	R	I	I	R	S	I	I	R	I	S	R	0.3
KP/20	S	S	S	R	I	S	S	R	R	I	R	R	0.4
Total R%	8(40%)	13(65%)	15(75%)	12(60%)	13(65%)	6(30%)	6(30%)	11(55%)	20(100%)	12(60%)	9(45%)	20(100%)	

Abbreviations are as follows: (S): Sensitive, (I): Intermediate, (R) Resistant, MAR: Multi-drug resistant, AK: Amikacin, ATM: Aztreonam, CIP: Ciprofloxacin, CAZ: Ceftazidime, Fep: Cefepime, CN: Gentamicin, IPM: Imipenem, LEV: Levofloxacin, SXT: Sulfamethoxazole/trimethoprim, TGC: Tigecycline, TZP: piperacillin/tazobactam, and LZD: Linezolid.

**Table 2:** EOP for phage ZCKP8 against *K. pneumoniae* isolates.

Bacterial Species	*K. pneumoniae* (*n* = 8)
EOP ≥ 1	4
EOP < 1	4

## Data Availability

Not applicable.

## Supplementary Materials

The following are available online at https://www.mdpi.com/article/10.3390/antibiotics10091048/s1, Table S1: Genome annotation of the ZCKP8 genome.
