# Supplementary material for: Topically Applied Bacteriophage to Control Multi-Drug Resistant Klebsiella pneumoniae Infected Wound in a Rat Model
Source: Antibiotics (Basel). 2021 Aug 27;10(9):1048. doi: 10.3390/antibiotics10091048 (PMC8470685; doi:10.3390/antibiotics10091048)
Supplement: Supplementary file 1 [file antibiotics-10-01048-s001.zip › antibiotics-1311635-supplementary.pdf]

antibiotics

MDPI

Article

Topically Applied Bacteriophage to Control Multi-Drug Resistant *Klebsiella pneumoniae* Infected Wound in a Rat Model

Mohamed S. Fayez [1], Toka A. Hakim [2], Mona M. Agwa [3], Mohamed Abdelmoteleb [4], Rania G. Aly [5], Nada N. Montaser [2], Abdallah S. Abdelsattar [1,6], Nouran Rezk [1] and Ayman El-Shibiny [1,7,*]

Table S1. Genome annotation of the ZCKP8 genome.

ORF number	Strand	Frame	CDS position	Translation	Best annotated protein
ORF1	+	3	312..497	186 | 61	Hypothetical protein
ORF2	+	3	513..791	279 | 92	Hypothetical protein
ORF3	+	3	798..1181	384 | 127	Hypothetical protein
ORF4	+	2	1178..1462	285 | 94	Hypothetical protein
ORF5	+	1	1315..1818	504 | 167	Hypothetical protein
ORF6	+	3	1818..1946	129 | 42	Hypothetical protein
ORF7	+	1	1894..2802	909 | 302	Hypothetical protein
ORF8	+	3	2895..3053	159 | 52	Hypothetical protein
ORF9	+	2	3053..3208	156 | 51	Hypothetical protein
ORF10	+	3	3489..3908	420 | 139	Hypothetical protein
ORF11	+	2	4028..5470	1443 | 480	Large terminase subunit
ORF12	+	2	5870..6109	240 | 79	Hypothetical protein
ORF13	+	1	6106..6258	153 | 50	Hypothetical protein
ORF14	+	3	6267..6455	189 | 62	Hypothetical protein
ORF15	+	2	6455..6676	222 | 73	Hypothetical protein
ORF16	+	1	6673..6873	201 | 66	Hypothetical protein
ORF17	+	1	6883..7128	246 | 81	Hypothetical protein
ORF18	+	3	7176..8570	1395 | 464	Portal (connector) protein
ORF19	+	1	8548..9360	813 | 270	Hypothetical protein
ORF20	+	2	9341..10288	948 | 315	head decoration protein
ORF21	+	1	11803..12240	438 | 145	Lysozyme
ORF22	+	3	12237..12482	246 | 81	Putative holin
ORF23	+	1	12457..12951	495 | 164	Tail protein
ORF24	+	2	12635..12904	270 | 89	Outer-membrane spanin protein
ORF25	+	3	14460..14675	216 | 71	Hypothetical protein
ORF26	+	2	14759..14950	192 | 63	Hypothetical protein
ORF27	+	1	14947..15171	225 | 74	Hypothetical protein
ORF28	+	3	15168..15404	237 | 78	Zinc-binding domain of primase-helicase
ORF29	+	1	15358..15864	507 | 168	Hypothetical protein
ORF30	+	3	15852..16142	291 | 96	Hypothetical protein
ORF31	+	3	16143..16487	345 | 114	Hypothetical protein
ORF32	+	2	16502..16654	153 | 50	Hypothetical protein
ORF33	+	2	16670..18070	1401 | 466	Hypothetical protein
ORF34	+	1	18070..18534	465 | 154	Capsid decoration protein
ORF35	+	3	18546..19634	1089 | 362	Major coat protein
ORF36	+	3	19677..19871	195 | 64	Hypothetical protein
ORF37	+	2	19901..20731	831 | 276	Hypothetical protein
ORF38	+	2	23456..23743	288 | 95	Hypothetical protein
ORF39	+	3	23652..24113	462 | 153	Hypothetical protein
ORF40	+	2	24110..24244	135 | 44	Hypothetical protein
ORF41	+	1	24232..24576	345 | 114	Hypothetical protein
ORF42	+	1	24580..24996	417 | 138	Hypothetical protein
ORF43	+	3	24993..25376	384 | 127	Hypothetical protein
ORF44	+	1	25828..25917	90 | 29	Hypothetical protein
ORF45	+	1	25990..26742	753 | 250	Major tail subunit
ORF46	+	1	28402..29109	708 | 235	Hypothetical protein
ORF47	+	3	29109..32402	3294 | 1097	Tail length tape measure protein
ORF48	+	2	32402..32872	471 | 156	Minor tail protein
ORF49	+	1	32872..33342	471 | 156	Hypothetical protein

ORF50	+	2	33305..33754	450 | 149	Tail assembly protein
ORF51	+	3	33702..36182	2481 | 826	Putative tail protein
ORF52	+	2	36212..38704	2493 | 830	Tail fiber protein
ORF53	+	1	45379..46332	954 | 317	DNA primase
ORF54	-	2	5781..5491	291 | 96	Hypothetical protein
ORF55	-	1	10423..10301	123 | 40	Hypothetical protein
ORF56	-	2	11286..10423	864 | 287	Hypothetical protein
ORF57	-	3	11414..11283	132 | 43	Hypothetical protein
ORF58	-	1	11704..11414	291 | 96	Hypothetical protein
ORF59	-	1	13225..13010	216 | 71	Hypothetical protein
ORF60	-	2	13359..13228	132 | 43	Hypothetical protein
ORF61	-	3	13580..13356	225 | 74	Hypothetical protein
ORF62	-	1	13969..13580	390 | 129	Hypothetical protein
ORF63	-	3	14363..14163	201 | 66	Holin
ORF64	-	2	20943..20761	183 | 60	Hypothetical protein
ORF65	-	3	21524..20946	579 | 192	DNA polymerase III beta subunit
ORF66	-	1	21655..21479	177 | 58	Hypothetical protein
ORF67	-	2	21999..21652	348 | 115	Hypothetical protein
ORF68	-	3	22850..21999	852 | 283	Putative transcriptional regulator
ORF69	-	2	22938..22822	117 | 38	Hypothetical protein
ORF70	-	3	23243..22935	309 | 102	Hypothetical protein
ORF71	-	1	25621..25523	99 | 32	Hypothetical protein
ORF72	-	2	27450..26782	669 | 222	Gp42
ORF73	-	3	27701..27450	252 | 83	Hypothetical protein
ORF74	-	1	28183..27698	486 | 161	HNH-endonuclease
ORF75	-	3	28319..28170	150 | 49	Hypothetical protein
ORF76	-	1	33457..33311	147 | 48	Hypothetical protein
ORF77	-	1	39481..38978	504 | 167	Single-stranded DNA-binding protein
ORF78	-	1	40528..39491	1038 | 345	Hypothetical protein
ORF79	-	3	41780..40824	957 | 318	Exonuclease
ORF80	-	1	42226..41783	444 | 147	Hypothetical protein
ORF81	-	2	42690..42274	417 | 138	Hypothetical protein
ORF82	-	1	44584..42677	1908 | 635	DNA helicase
ORF83	-	1	45331..45179	153 | 50	Hypothetical protein
ORF84	-	2	45366..44593	774 | 257	Putative endonuclease protein
ORF85	-	1	46579..46346	234 | 77	Hypothetical protein
ORF86	-	2	48132..46576	1557 | 518	Hypothetical protein
ORF87	-	3	48365..48129	237 | 78	Hypothetical protein
